# Natural Killer Cell-Based Immunotherapy in Gynecologic Malignancy: A Review

**DOI:** 10.3389/fimmu.2017.01825

**Published:** 2018-01-05

**Authors:** Locke D. Uppendahl, Carly M. Dahl, Jeffrey S. Miller, Martin Felices, Melissa A. Geller

**Affiliations:** ^1^Department of Obstetrics, Gynecology and Women’s Health, Division of Gynecologic Oncology, University of Minnesota School of Medicine, Minneapolis, MN, United States; ^2^University of Minnesota School of Medicine, Minneapolis, MN, United States; ^3^Department of Medicine, Division of Hematology, Oncology, and Transplantation, University of Minnesota School of Medicine, Minneapolis, MN, United States

**Keywords:** natural killer cells, immunotherapy, gynecologic malignancy, ovarian cancer, cervical cancer

## Abstract

Harnessing the immune system has proven an effective therapy in treating malignancies. Since the discovery of natural killer (NK) cells, strategies aimed to manipulate and augment their effector function against cancer have been the subject of intense research. Recent progress in the immunobiology of NK cells has led to the development of promising therapeutic approaches. In this review, we will focus on the recent advances in NK cell immunobiology and the clinical application of NK cell immunotherapy in ovarian, cervical, and uterine cancer.

## Introduction

Exploiting the immune system has proven an effective therapeutic approach in treating a variety of malignancies. Identified in 1975, natural killer (NK) cells exist in the blood as preactivated cytolytic lymphocytes and are recognized as the most efficient antitumor effector (1–3). Distinct from T and B cells, NK cell effector function is not mediated by high-resolution antigen specificity but through signaling of multiple germ line-encoded activating and inhibiting receptors. Over the past 40 years, research has defined the regulation of NK cells and established essential roles they play in anticancer immunity. Strategies to harness and augment NK cells for cancer therapy are a relatively new and rapidly developing field. At this point, the success of NK cell-based immunotherapy has largely been confined to hematologic malignancies and has yet to translate to solid organ tumors (4). As a subset of solid organ tumors, gynecologic malignancies are a heterogeneous group of tumors derived from vulvar/vaginal, cervical, uterine, fallopian, and ovarian tissues. The treatment regimens for gynecologic cancers continue to develop with great room for improvement. With increased understanding of NK cell biology, there is renewed interest in NK cell-based immunotherapy directed against gynecologic malignancies. In this review, the advances in our understanding and clinical application of NK cell immunotherapy against ovarian, cervical, and uterine cancer is summarized.

## Characteristics of NK Cells

The innate and adaptive immune systems function together to recognize and effectively eliminate aberrant cells, including cancer. Historically seen as part of the innate immune response, NK cells are large, granular lymphocytes. They were found to have the ability to kill tumor cells without any prior sensitization (thus “natural”) or restriction of major histocompatibility complex (MHC) molecule expression (1, 2, 5).

Phenotypically, NK cells are defined *via* flow cytometry by the absence of CD3 and the presence of CD56 surface expression and comprise approximately 5–10% of circulating lymphocytes (6, 7). NK cells can be divided into CD56^bright^CD16^−^ or CD56^dim^CD16^+^ populations with different functional properties. Developmentally immature CD56^bright^CD16^−^ NK cells are capable of producing abundant cytokines, particularly interferon gamma (IFN-γ) and tumor necrosis factor alpha (TNF-α), immediately after activation but possess little direct cytolytic function. In contrast, mature CD56^dim^CD16^+^ NK cells are characterized by the direct killing of transformed cells *via* perforin/granzyme release or death receptor pathways (Fas, TNF-related apoptosis-inducing ligand, TRAIL) (8–10).

As discussed below, NK cells are involved in tumor immunosurveillance and mediate antitumor responses (11). Their activity is highly regulated by a variety of germ line-encoded inhibitory and activating receptor expression (12, 13). Collectively, the complex balance of inhibitory and activating signals promotes self-tolerance or drives potent effector function of NK cells.

## NK Cell Effector Functions

Natural killer cells identify and eliminate foreign, infected, damaged, or malignant cells through a variety of mechanisms. The most well-known is through receptor-mediated cytotoxicity. NK cells express a series of activating receptors capable of binding stress-induced ligands expressed on tumor cells. They also express a number of inhibitory receptors that interact with ligands to induce activation-limiting signals. When activating signals over-ride inhibitory mechanisms, the NK cell mediates exocytosis of stored lytic molecules. The membrane-disrupting protein perforin and serine protease granzymes then function in coordination to gain access to the target cell and induce apoptosis through the activation of caspases (14, 15).

Natural killer cells also appear to be the principal effectors for a process called antibody-dependent cell-mediated cytotoxicity (ADCC) (16). ADCC occurs when targets that become coated by antibody are recognized by NK cells *via* ligation to the low-affinity receptor for the Fc portion of human immunoglobulins, CD16 (FcγRIIIa). Upon binding, downstream signal transduction mechanisms lead to NK cell degranulation, cytokine secretion, and tumor cell lysis (15). The recent advances in our understanding of ADCC and NK function can be applied to augment NK cell immunotherapy. For example, monoclonal antibodies (mAbs) targeting CD20 (rituximab), Her2/neu (herceptin), epidermal growth factor receptor (cetuximab and panitumumab), and disaloganglioside (GD2) demonstrate significant antitumor contributions from NK cell-dependent ADCC in addition to the direct antitumor effect of the antibody (17). This strategy maintains the specificity against key molecular tumor targets important for cell proliferation or tumor growth with the added contribution of ADCC *via* NK cell effector function (16). Highlighting this role of ADCC, previous studies have demonstrated depletion in NK cell populations decreases the efficacy of mAb therapy (18). There is further evidence showing that specific FcγR polymorphisms impact responsiveness to mAb therapy and may even predict clinical outcomes for certain tumors (19–22). Today, mAb are being developed with enhanced affinity for CD16 to better activate NK cells and improve antitumor response (23, 24). Unfortunately, strategies to include the use of mAb to enhance ADCC in gynecologic malignancies have not been thoroughly investigated.

Natural killer cells can also initiate the transduction of death signals within target cells through death ligand/receptor ligation (25). NK cells are capable of expressing Fas ligand or TRAIL (26, 27). Interaction of these ligands with their respective antigens on tumor cells activates caspases and induces apoptosis (14). Recent studies have demonstrated the proteasome and histone deacetylase inhibitors upregulate the expression of death receptors and enhance NK cell-mediated cytotoxicity of tumor cells through the death receptor pathways (28–30). This is particularly interesting because this strategy was effective in both hematologic and solid tumors.

**Figure 1:**
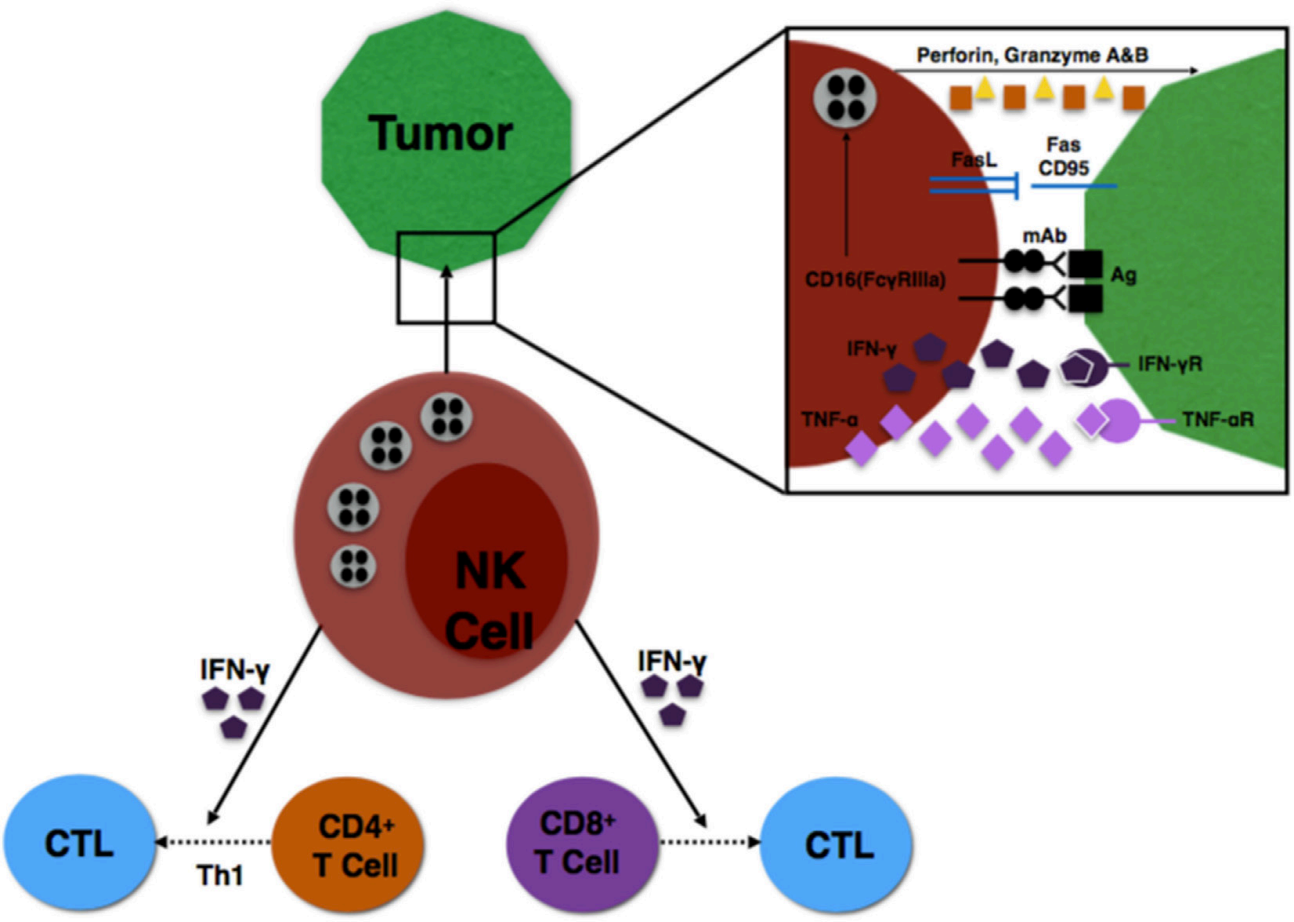
Illustration of NK cell interaction with tumor cells, as well as NK influence on T cell differentiation.

Finally, specific subsets of NK cells are capable of producing important immunoregulatory cytokines (31). NK cells expressing CD56^bright^ are the primary source of NK cell-derived IFN-γ, TNF-α, and other cytokines that play a major role during the innate immune response to infection or tumorigenesis (8). The NK cells provide an early source of IFN-γ to induce CD8^+^ T cells to become cytotoxic T lymphocytes (CTLs) and drive a Th1 response of CD4^+^ T cells to further promote CTL differentiation (32, 33). These interactions are illustrated in Figure 1.

## Important NK Cell Receptors

Due to the capability of immediate response, NK cells are tightly regulated through a combinatorial array of surface receptors. Functionally, these receptors are classified as activating or inhibitory with their ligands either members or homologs of MHC class I molecules. Structurally, they belong either to the immunoglobulin (Ig)-like receptor superfamily or the C-type lectin-like receptor (CTLR) superfamily (34). We will review a few of the selected inhibitory and activating receptors on human NK cells.

The NK cell response is dominated by a variety of germ line-encoded inhibitory receptors from three families: killer immunoglobulin-like receptors (KIRs), C-type lectins (including NKG2A-CD94), and leukocyte immunoglobulin-like receptors (35–37). Stochastic surface expression within these families leads to subsets of NK cells with a diverse repertoire of receptors (35). Ligands for these receptors are both “classical” and “non-classical” class I molecules encoded within the MHC, termed human leukocyte antigens (HLAs) (38–40). For example, inhibitory KIRs recognize the classical HLA-A, HLA-B, and HLA-C proteins but do not distinguish self from non-self peptides. The binding of inhibitory KIR on NK cells to their HLA cognate suppresses cytotoxicity and cytokine secretion. The diverse groups of inhibitory receptors are all glycoproteins that signal through the canonical immunoreceptor tyrosine-based inhibitory motif (ITIM) to suppress NK cell response. Only when sufficient activating signals are present does the NK cell initiate effector function.

The *CD94* and *NKG2* family of genes encode CTLRs that recognize non-classical MHC class I molecules (HLA-E, -F, -G, and -H) and play a dominant role in NK cell function. CD94 can heterodimerize with NKG2A and signal through ITIM to function as an inhibitory receptor when bound to HLA-E. However, CD94/NKG2C heterodimers serve as activating receptors. The inhibitory CD94/NKG2A and the activating CD94/NKG2C receptors are found in overlapping subsets of NK cells in the peripheral blood (38). Not surprisingly, CD94/NKG2A inhibitory receptor binds HLA-E with higher affinity compared to CD94/NKG2C activating receptor (41).

The role and function of NK inhibitory receptors are well defined. Recent research has elucidated the NK effector functions of activating and coactivating receptors. Unlike B and T cells, NK cells do not possess an activating receptor that dominates their development and effector function. Instead, they express a complement of invariant activating receptors that include the NK specific natural cytotoxicity receptors (NCRs) (NKp46, NKp30, and NKp44), C-type lectin-like (NKG2D, CD94/NKG2C), 2B4, DNAM-1, NTB-A, NKp80, CD59, and CD16 among others (36, 38, 42, 43).

The *NKG2D* gene encodes one activating receptor that, unlike its name, shares very little homology with *NKG2A* or *NKG2C*. NKG2D is a type II transmembrane-anchored glycoprotein constitutively expressed on all human NK cells and recognizes cell surface glycoproteins structurally related to MHC class I molecules. NK cells stimulated through NKG2D initiate cell-mediated cytotoxicity and cytokine release. Known human ligands include MICA, MICB, ULBP1, ULBP2, ULBP3, and ULBP4 and are upregulated by stress and stalled DNA replication *via* DNA-damage checkpoint pathways (34, 44). MICA and MICB are stress-induced antigens frequently expressed by tumors (45). However, progressive stages of cancer are associated with tumor shedding of MICA/B, which appears to systemically impair the immunological competence of individuals with cancer by causing downregulation of NKG2D. This impairment of effector function promotes tumor immune evasion (46, 47). Targeting the tumor pathways that lead to the upregulation of NKG2D ligands or alternatively maintain and/or upregulate NKG2D receptors may be a productive method to enhance NK cell-based immunotherapy.

The activating DNAM-1 receptor (CD226) belongs to the Ig superfamily and is constitutively expressed on all human NK cells (48). The specific ligands CD112 (nectin-2) and CD155 (polio virus receptor) bind and augment NK cell-mediated cytotoxicity and cytokine release (49, 50). Similarly to MICA, ovarian cancer cells ubiquitously expressing the ligand CD155 show reduced DNAM-1 expression and impaired NK cell function (51). As our understanding of these signaling and inhibitory pathways expands, our potential targets for NK immunotherapy grow.

Other triggering receptors are the NCRs, which include NKp46 (NCR1, CD335), NKp44 (NCR2, CD336), and NKp30 (NCR3, CD337) (52–56). These receptors have a variety of ligands with various structures. NKp46, the main activating receptor for human NK cells, binds the hemagglutinins on influenza virus-infected cells (57). The human cytomegalovirus pp65 tegument protein was identified as the original ligand for NKp30 and was shown to be responsible for suppression of NK cell cytotoxicity (58). Later on, HLA-B associated transcript 3 protein (BAG6) and B7-H6 (NCR3LG1) were identified as novel surface ligands of NKp30 (59–61). Importantly, B7-H6 is present on a broad spectrum of tumors and may play a role in antitumor immunity (62). In addition, tumor shedding of B7-H6 was demonstrated to be a novel mechanism of immune escape (63). A recent study in patients with ovarian cancer demonstrated B7-H6 tumor ligands were associated with decreased NKp30 expression of tumor-associated NK cells (64). These NK cells demonstrated impaired IFN-γ production and cytolytic function. Together, these findings indicate how NK cells may recognize and kill target cells without the decreased expression of MHC class I protein and serve as a template for designing molecules to stimulate NK-mediated cytotoxicity for tumor immunotherapy. It also exemplifies the important role the tumor microenvironment plays on NK cell function and immune surveillance, which will be elucidated below.

## NK Role in Immunosurveillance

In 1909, the German physician Paul Ehrlich predicted the immune system routinely identified and eliminated aberrant cells that would otherwise lead to cancer (65). The theory was revisited with great interest 50 years later following a deeper understanding of tumor immunobiology (66–70). Eventually, the concept of tumor immunosurveillance was experimentally validated with the advancement in mouse genetics and the generation of mAb production (71–73). The complex relationship between the tumor and immune system further expanded to incorporate tumor immunoediting, a process where tumor cells reduce their immunogenicity thereby rendering the immune system incapable of recognizing and destroying the aberrant cells (74). Today, it is accepted both innate and adaptive immunity play vital roles in continuously monitoring tissues to eliminate aberrant tumor cells (73).

Some of the earliest experimental evidence detailing the role NK cells play in tumor control and immunosurveillance was obtained in mice. An early study demonstrated beige (bg) mice with 75 NK cell activity resulted in increased tumor growth rate and metastasis compared to control mice with normal NK cell function (75). The presence of NK cell activity also correlated with better control of *in vivo* tumor growth and metastasis, particularly against histocompatibility complex (MHC) class I-deficient variants (76). In addition, mice depleted of NK cells by the anti-asialo-GM_1_ mAb resulted in a twofold to threefold increase in 3′-methylcholanthrene (MCA)-induced tumorigenesis compared to wild-type controls (77). Similarly, mice with defective NK function due to a deficiency of NK1.1^+^CD3^−^ cells but with functionally normal B, T, and NK/T cells showed impaired *in vivo* rejection of tumor cells (78).

More recently, a prospective cohort of 3,625 individuals were assessed for natural cytotoxic activity of peripheral blood lymphocytes and then followed for 11 years to observe the incidence of cancer (79). These results indicate individuals with impaired NK cell function display an increased risk of developing cancer. In an alternative study, NK cells were identified to also play a role in surveillance against DNA damage through checkpoint pathways (44). The DNA damage response is activated early in tumorigenesis and induces surface expression of ligands for an activating receptor of NK cells, providing a link between the innate immune system and tumor surveillance (80). Together, these studies highlight the contribution NK cell effector function plays in immune protection from tumor development.

It is now established that NK cells participate in first-line defense against tumor development. NK cells are able to discriminate self and non-self due to a wide array of cell surface receptors that control their response, particularly the MHC class I molecules (38). In a 1986 observation, NK cells eliminated MHC class I-deficient cells but not cells with normal MHC class I expression (81). This seminal observation was termed the “missing-self” hypothesis; in the absence of critical surface proteins cells are recognized and eliminated by NK cells (82, 83). This feature is important because solid tumors undergoing malignant transformation frequently reduce MHC class I expression and this process represents one of the main mechanisms for tumor cells to avoid detection by the adaptive immune system (84–88). Thus, one key function of NK cells is to monitor the integrity of MHC class I expression on tumor cells. The significance uncovered in the “missing-self” hypothesis was NK cell effector function is actively inhibited with engagement of MHC class I molecules on cells. More importantly for solid tumor malignancies, newer evidence suggests inhibitory MHC class I receptors only dampen, rather than eliminate, the effector function of NK cells (89–91). This suggests a sufficient activating signal either through a single potent stimulation or the simultaneous engagement of multiple activating receptors is capable of mediating NK cell effector function to eliminate target cells despite MHC class I expression. Thus, a modified “missing-self” hypothesis states “NK cells patrol for abnormal cells that lack MHC class I or overexpress ligands for activating NK cell receptors” (38).

## The Tumor Microenvironment and Its Impact on NK Function

The malignant transformation of normal cells results from a multifactorial process resulting in genomic instability and a modification of immunosurveillance mechanisms that induce tolerance (92). As tumors evolve, they develop different strategies to escape the immune response: (i) the secretion of immunosuppressive cytokines or soluble tumor-derived inhibitory factors, (ii) the expression of co-inhibitory or loss of co-stimulatory receptors, and (iii) the loss or downregulation of MHC class I molecules (84). To be effective, NK cells must first extravasate through the vessel endothelial lining and migrate to the tumor tissue (93). Any localizing defect can lead to insufficient numbers of NK cells to the primary or metastatic sites. Once in the extravascular space, NK cells encounter hypoxia, acidic pH, and low glucose conditions that are hostile to immune effector cell function (94). Peripheral blood NK cells and tissue NK cells are notably different; tumor NK cells are functionally defective, incompletely activated, or anergic compared to peripheral blood effectors. In addition, the effectors found within solid tumors are often in limited supply. Further understanding the mechanisms of tissue immunity and its impact on NK cells will be important in our ability to treat solid tumors therapeutically with immunotherapy.

Here, we will discuss how the tumor microenvironment limits the effectiveness of the NK cell antitumor response with attention to ovarian cancer, cervical cancer and endometrial cancer.

### Epithelial Ovarian Carcinoma

Ovarian cancer is the most lethal gynecologic malignancy, with an estimated 14,080 deaths expected in the United States for 2017 (95). Despite optimal treatment with surgery and adjuvant chemotherapy, the recurrence rate approaches 70–80% (96). Although the disease tends to remain confined to the abdominal cavity, women with recurrent ovarian cancer progress and ultimately die. Therefore, there is an urgency to develop new and effective therapies.

Important to effective therapeutic development is the understanding of the immunologic interactions within the tumor and its physiologic impacts, which often includes the development of profuse ascites. It is well documented that the ascites fluid from patients with advanced stage ovarian cancer suppress the function of otherwise normal immune effectors, including NK cells (97–101). The ascites contains large numbers of growth factors and cytokines that promote the proliferation of tumor cells (102–104). While fresh NK cells (CD56^+^CD3^−^CD16^+^) isolated from the ascites fluid are found in relatively high concentrations compared to peripheral blood, they are functionally deficient (105, 106). This population of NK cells demonstrates decreased CD16 expression and have reduced proliferative, cytolytic, and cytokine production compared to peripheral blood NK cells (107). Signaling proteins vital to interpreting the activating and inhibitory signals become defective and alter the expression of cytokine transcripts and proteins (100). As detailed above, tumor-associated ligands MICA/B and B7-H6 are often found within the peritoneal fluid of serous ovarian cancer patients and impair NK cell effector function (46, 64). These deficiencies act in combination and likely influence the ability to control the spread and proliferation of tumor cells within the peritoneal cavity of patients with advanced ovarian cancer.

For ovarian cancer, the role of the immune response has been well documented with immunohistochemistry. Multiple studies document a positive correlation between the number of tumor-infiltrating lymphocytes within the tumor and overall survival (OS) (108–110). The absence of CTL infiltration (CTL) also predicts platinum resistance (111). More recently, genomic profiling studies also support using immunophenotype as a method to predict response to therapy and clinical outcomes (112–114). However, most of the published studies document limited infiltration of NK cells within the primary ovarian tumor and cells that suppress immune response and support tumor growth dominate (108, 115–120). The presence of infiltrating NK cells impact on OS is also controversial. Infiltrating NK cells have largely not been associated with better outcomes, and in one case, predicted worse OS (121). However, it was recently shown that CD103^+^ tumor-infiltrating NK cells were almost always found with CD8^+^ T cells and were the second best predictor of positive outcomes in primary ovarian cancer (122). In light of this evidence, further study regarding the role of infiltrating NK cells is warranted. Regardless of the uncertainty of the role of NK cells and OS, freshly isolated NK cells from the tumor are drastically impaired through a variety of mechanisms (123, 124).

Within the tumor tissue, NK cells have complex interactions with other immune cells with suppressive functions, including myeloid-derived suppressor cells (MDSCs) and regulatory T cells (T_reg_) (125). The MDSCs typically suppress effector function of T cells, thereby promoting tumor growth. In mice, a subset of MDSCs expresses the NKG2D ligand Rae-1 and is capable of elimination through NK cell-mediated cytolysis (126). If present, NK cells are also potent producers of IFN-γ and prevent the macrophage polarization toward the M2 phenotype that support tumor progression (127).

Another mechanism interfering with NK effector function within the microenvironment is the secretion of immunosuppressive cytokines. The hypoxic environment induces transcription of interleukin (IL)-8, a chemokine important for tumor growth, angiogenesis, and metastasis (128–130). Women with ovarian cancer have significantly elevated concentrations of IL-8 compared to benign controls (131). In addition to promoting angiogenesis and tumor growth, *in vitro* studies demonstrate an immunosuppressive effect of IL-8 by inhibiting TNF-induced apoptosis (132). In addition, the cytokine transforming growth factor-β (TGF-β) can contribute to the immunosuppressive microenvironment (133). Overproduction of TGF-β by tumor cells suppresses CD16-mediated NK cell IFN-γ production and ADCC (134). These immunosuppressive cytokines attenuate NK cell effector function and limit the antitumor response.

### Cervical Carcinoma

Cervical cancer is a human papillomavirus (HPV)-induced cancer. Mediated by an adaptive immune response against viral proteins, the greatest success involving immunotherapy in gynecologic malignancies is the development of vaccines against HPV and prevention of cancer (135). The two early viral proteins E6 and E7 are defined tumor-associated antigens and are processed and expressed on MHC class I molecules. However, HPV-induced cervical cancers often show altered expression of MHC class I molecules resulting in the inability of CTLs to recognize the peptide epitopes (88, 136–138). The HPV16 E6 and E7 oncoproteins also inhibit NK cell IL-18-induced IFN-γ production likely contributing to viral pathogenesis (139). In addition, HPV infection is non-lytic and produces only a modest inflammatory infiltrate of macrophages and lymphocytes (140). These changes likely affect the efficacy of the innate immune response and provide opportunities to escape immune surveillance. However, the “loss of self” may render these HPV-related cancers susceptible to NK cell attack.

Very little is known in regard to NK cell function and phenotype in women with cervical cancer. Freshly isolated peripheral blood NK cells in women with cervical cancer and benign healthy controls demonstrated no significant functional differences until the patient had distant metastatic disease (stage IVb) (141). Another study demonstrated infiltrating NK cells of patients with cervical cancer were present and of the CD56^bright^CD16^−^ phenotype. They also observed the upregulation of the DNAM-1 ligand CD155 and the NKG2D ligand MICA in cervical cancer but not in cervical intraepithelial neoplasia or normal controls (142). A follow-up study demonstrated keratinocyte expression of HPV16 E6 and E7 produced rapid induction of intracellular adhesion molecule-1 protein levels (143). NK cells recognize expression of these ligands and adhesion molecules and may be a promising strategy to target for the treatment of cervical cancer.

Interestingly, certain combinations of KIRs and HLA loci associated with NK cell activation increase the risk of developing cervical cancer (144). Specifically, the presence of the activating KIR3DS1 on NK cells in the absence of ligands for inhibitory KIRs results in an increased risk of cervical neoplasia. In constrast, NK cell effector inhibition mediated by KIR2DL1 and KIR3DL1 in the absence of KIR3DS1 results in protection from cervical neoplasia. KIR receptors and HLA ligands interact through an epistatic relationship in which HLA ligands activate a genetic molecular cascade through the KIR receptor that influences NK functionality. This KIR/HLA interaction suggests that an inappropriate inflammatory response, mediated by NK cell KIR-ligand interactions, may lead to tumor progression. The precise role of NK cells in the context of cervical cancer is far from being defined.

### Endometrial Carcinoma

Endometrial carcinoma arises from the lining of the uterine cavity and is the fourth most common malignancy in women (95). Most women are diagnosed with low-grade, early stage disease and are cured following surgery. There is very little information on the microenvironment of uterine NK (uNK) cells and cancer. There is much more known about NK cell biology and pregnancy.

Uterine NK cells are a tissue-specific, specialized population of cells that make up large percentage of both endometrial and decidual lymphocytes (145). uNK cells are almost exclusively CD56^bright^CD16^−^, although they contain cytotoxic granules (146). uNK cells are thought to contribute to immunosuppressive mechanisms during pregnancy when immune tolerance is vital. As a result, these uNK cells also display less cytotoxicity against tumor targets compared to peripheral NK cells (147). uNK cells are not only immunosuppressive during pregnancy in order to protect the fetus but also may play a key role in modulating fetal growth, with activated uNK cells at the maternal–fetal interface producing factors that play a role in the regulation of trophoblast invasion and uterine vascular remodeling. These roles are critical to placental formation and healthy gestation (148, 149). A number of interesting questions remained unanswered with respect to NK cells and uterine cancer: (i) does the immunosuppressive nature of the maternal–fetal interface contribute to developing uterine carcinoma? (ii) Do peripheral NK cells migrate into the uterine cavity with tumorigenesis? (iii) Are uterine tumor cell lines susceptible to NK cell killing? More complete knowledge of the biology and function of uNK cells in endometrial cancer is required prior to developing strategies of NK cell immunotherapy for this malignancy.

## NK Immunotherapy in Gynecologic Malignancies

Advances in understanding the NK cell biology and function over the last few decades have resulted in promising new immunotherapeutic approaches for gynecologic malignancies, in particular ovarian cancer. There is scant research published on NK cell-based immunotherapy in cervical and uterine carcinoma at this time. In this section, we will review recent advances in NK cell-based immunotherapy for all gynecologic malignancies highlighting the opportunities and challenges for each cancer.

### Early Trials Using Biologic Response Modifiers and Cytokine Therapy

Early clinical trials in ovarian cancer patients aimed to improve the antitumor activity of immune cells through intraperitoneal injection of biological response modifiers, including *Corynebacterium parvum*, bacillus Calmette-Guerin, leukocyte interferons (IFN), and irradiated autologous and allogeneic tumor cells (150). Overall, these agents had limited success in treatment response with relative toxic side effects. Another novel strategy using an attenuated strain of influenza virus to infect ovarian cancer tumor cell lines was later developed. The nonviable extracts from the tumor cells, termed viral oncolysate, were isolated and then injected intraperitoneally (IP) into patients with ovarian cancer with both clinical and pathological responses noted (151, 152). Follow-up studies noted the viral oncolysate enhanced the NK cell response (153). Although better tolerated, the treatment had limited clinical responses.

Advancement in recombinant DNA technologies led to the purified production of cytokines and their use to treat a variety of malignancies. Today, cytokines are easy to manufacture and administer. While the central goal of cytokine therapy is to potentiate the autologous antitumor response *in vivo*, they lack specific immunomodulatory effects (154). Table 1 lists selected clinical trials in ovarian cancer evaluating cytokine therapy and NK cell response if reported. The first generation of cytokines were recombinant (r) IFN-α, rIFN-γ, and rIL-2. The published results of IP therapy with recombinant IFN with or without chemotherapy have been comprehensively reviewed by Freedman and colleagues (155). They also reported on eight clinical trials evaluating IP therapy with IL-2 alone or in combination with cellular therapy (discussed below). The results demonstrated IP immunotherapy with cytokines was tolerated but had varying levels of success. Two important features were identified: (i) response was dependent on remaining tumor burden prior to initiation of therapy and (ii) efficacy of first-line therapy is critical in these patients. Most of these early trials did not assess NK cell response to therapy.

**Table 1:** Natural killer (NK) cell findings from clinical trials of cytokine immunotherapy for ovarian cancer.

Year	No. of patients	Population	Treatment	Phase	Clinical response	NK cell response	Reference
1982	5	Recurrent disease	IM interferons (IFN)	I	1 patient had PR; 2 patients with SD at 12 months	Increase in NK cell activity in peripheral blood in all three patients examined	Einhorn et al. (192)
1984	14	Persistent disease EOC on second look laparotomy	Intraperitoneally (IP) IFN-α	I	11 pts underwent surgical reevaluation after therapy: 4 had CR (36%), 1 PR (9%), and 6 with disease progression (55%)	NK cytotoxicity was elevated in the IP cavity	Berek et al. (98)
1987	40 (1 OvCa)	Advanced cancer	Continuous IL-2	I	PR in 1 OvCa pt	NR	West et al. (193)
1996	108	Persistent disease EOC on 2nd look laparotomy	IP IFN-γ	II	98 evaluable pts: 31 (32%) with surgical RR, including 23 (23%) with CR	NR	Pujade-Lauraine et al. (194)
2000	148	First-line therapy	Cisplatin/cyclophosph amide ± SubQ IFN-γ	III	Progression-free survival (PFS) at 3 years: 38% in control to 51% in treatment; CR 56% in control vs 68% in treatment; similar toxicity	NR	Windbichler et al. (195)
2005	44	Maintenance following second-line	Low-dose SubQ IL-2 + RA	II	Treatment decreased VEGF and improved immune function; absolute difference of 42% between treatment and matched controls in both PFS and overall survival (OS) at 2 years; well tolerated	Treatment improved lymphocyte and NK cell counts	Recchia et al. (159)
2008	847	First-line therapy	Carboplatin/paclitaxel ± SubQ IFN-γ	III	Stopped early due to interim analysis: shorter OS (60 vs 70%) in pts receiving IFN-γ at time of analysis; more adverse events	NR	Alberts et al. (156)
2009	31	Platinum-resistant/refractory disease	IP IL-2	II	24 evaluable pts: 6 (25%) with surgical RR, including 4 CR (17%); well tolerated	NR	Vlad et al. (196)
2010	65	Maintenance following second-line	Low dose SubQ IL-2 + RA	II	Overall RR 57%, including 4 (6%) CR; median PFS 23.2 months and median OS 52.8 months	Treatment improved NK cell counts and decreased VEGF	Recchia et al. (158)

Recently, a randomized phase III trial of 847 women with stage III or IV ovarian cancer evaluating front-line combination carboplatin/paclitaxel plus subcutaneous *Escherichia coli*-derived recombinant IFNγ-1b was stopped early following a second interim analysis (156). At the time of analysis, patients receiving IFNγ-1b plus chemotherapy compared to chemotherapy alone demonstrated significantly shorter OS (60 vs 70%). It should be noted IFNγ-1b has biological activity identical to natural human IFN-γ (157). The authors speculate IFNγ-1b may have resulted in activation of T_regs_ and immunosuppression. They also suggest treatment with IFNγ-1b is more toxic and leads to decreased treatment adherence or dose reductions of chemotherapy. The ability to complete all six cycles of chemotherapy was compromised in patients receiving IFNγ-1b (77 vs 83%). Regardless, it was concluded that IFNγ-1b does not have a role in first-line treatment of advanced ovarian cancer.

Finally, a phase II trial of advanced ovarian cancer patients treated with second-line therapy followed by maintenance low-dose subcutaneous IL-2 with oral 13-cis-retinoic acid reported an overall response rate of 57% (158, 159). Treatment was associated with an improvement of peripheral NK cell counts and a decrease in VEGF compared to baseline. In this cohort of 65 patients, the progression-free survival (PFS) and OS was 29 and 38%, respectively.

Similar to IL-2, the cytokine IL-15 can potently increase NK cell numbers. While IL-2 and IL-15 share common signaling mechanisms, they differentially control the development, activation, and proliferation of NK cells (160). IL-2 activates a broad range of T cells, including T_regs_. In contrast, IL-15 preferentially stimulates CD8^+^ T cells and non-terminally differentiated NK cells and has been shown to enhance NK cell function in the ovarian cancer setting (101, 161). Several clinical trials evaluating IL-15 are underway (162).

In summary, biologic response modifiers and cytokine therapy have demonstrated conflicting results in the limited clinical trials. In addition, the small number and heterogeneity of study participants limit interpretations. Additional investigations examining the role of cytokines in ovarian cancer and reporting standard immune and clinical responses is warranted.

### Adoptive Transfer of NK Cells

Initial efforts in adoptive transfer of immune cells aimed to improve the autologous antitumor responses through cytokine stimulation (155, 163–165). Immune cells removed from the peripheral blood of patients were activated with various cytokines and then infused back into the same patient. Table 2 lists selected clinical trials in ovarian cancer evaluating adoptive transfer of NK cell-related therapy and response if reported.

**Table 2:** Natural killer (NK) cell findings from clinical trials of adoptive cellular transfer for ovarian cancer.

Year	No. of patients	Population	Treatment	Phase	Clinical response	NK cell response	Reference
1989	20 (7 OvCa)	NR	Autologous IP lymphokine-activated killer (LAK) + IL-2	I	2/7 OvCa pts had PR; extended therapy was hampered by IP fibrosis	NR	Urba et al. (165)
1990	24 (10 OvCa)	Recurrent disease	Autologous IP LAK + IL-2	I	2/10 laparoscopic documented PR; 8/10 no response; progressive IP fibrosis	NR	Steis et al. (166)
1990	10	Recurrent disease	Autologous IP LAK + IL-2	I	1/10 (10%) RR; dose-limiting toxicity was ascites accumulation	LAK activity correlated with CD3^−^CD56^+^ lymphocytes	Stewart et al. (167)
1991	7	Advanced or recurrent disease	Cyclophosphamide, ACT of tumor-infiltrating lymphocytes (TIL)	II	5/7 (71%) had RR, including 1 (14%) CR	NR	Aoki et al. (197)
	10		Cisplatin, ACT of TIL		9/10 (90%) had RR, including 7 (70%) CR		
2011	20 (14 OvCa)	Refractory disease (4+ prior therapies)	Allogeneic IV NK + IL-2	II	Well tolerated overall, but 2 severe adverse events including 1 death; 4/14 (29%) OvCa pts had RR, 8/14 (57%) with SD, and 1/14 (7%) with PD	No sustained *in vivo* expansion of NK cells was noted	Geller et al. (173)
2014	92	First-line therapy	Primary debulking surgery, carboplatin/paclitaxel ± autologous IV cytokine-induced killer (CIK) cells	III	Progression-free survival: 37.7 vs 22.2 months favor CIK (*p* = 0.004); overall survival 61.5 vs 55.9 months (NS); well tolerated	NKT (CD3^+^CD56^+^) cells increased; NK cells decreased in CIK culture; no changes in peripheral NK cells	Liu et al. (171)
2016	20 (2 OvCa)	Advanced or recurrent disease	Allogeneic IV NK	I	Well tolerated; 1 had SD and 1 had PD	*Ex vivo* expanded and activated NK cells were generated and safely administered	Yang et al. (174)
2017	1	First-line therapy	Allogeneic IV NK	Case report	PR, with CA-125 decreasing 11,270 to 580 after 6 treatments	Expanded NK cells in culture	Xie et al. (175)

The early phase I clinical trials evaluating the adoptive transfer of autologous lymphokine-activated killer (LAK) cells with high-dose IL-2 therapy demonstrated limited clinical responses with high rates of peritoneal fibrosis (165–167). Similar to LAK immunotherapy, cytokine-induced killer (CIK) cells arise from peripheral blood mononuclear cell cultures with stimulation of anti-CD3 mAb, IFN-γ, and IL-2 (168). CIK cells are characterized by a mixed T-NK phenotype (CD3^+^CD56^+^) and demonstrate enhanced cytotoxic activity compared to LAK cells against ovarian and cervical cancer (169, 170). A recent phase III clinical trial investigated adoptive transfer of autologous CIK cells following primary debulking surgery and adjuvant carboplatin/paclitaxel chemotherapy (171). Advanced epithelial ovarian cancer patients (*n* = 92) were paired to receive maintenance monthly CIK transfusions (*n* = 46) vs standard of care observation (*n* = 46). Median PFS was 37.7 months in the treatment group and 22.2 months in the control group (*p* = 0.004). Median OS in the treatment group was 61.5 months, compared to 55.9 months in the control group (*p* = 0.289). The therapy was well tolerated with no grade III or IV adverse reactions. Interestingly, the proportion of T_regs_ in peripheral blood decreased following two courses of immunotherapy (*p* = 0.006). While only a small, non-randomized phase III study, these results are promising and follow-up studies are warranted.

Recently, insights into the molecular mechanisms regulating NK cell function shifted the focus toward allogeneic NK cell immunotherapy. The mismatch between donor KIR repertoire and recipient MHC class I molecules can improve the antitumor activity of NK cells (172). In a phase II clinical trial, we studied haplo-identical related IV infused NK cells in patients with recurrent ovarian (*n* = 14) and breast cancer (*n* = 6) (173). Following a lymphodepleting chemotherapy regimen ± radiation, women received adoptive transfer of a CD3/CD19-depleted NK cell product and were treated with subcutaneous IL-2 injections. No successful NK cell persistence or expansion was noted, likely as a result from recipient T_reg_ expansion and reconstitution following therapy. Only two other small reports using allogeneic NK cell therapy in ovarian cancer have been published (174, 175). Together, these studies suggest allogeneic NK cell therapy is feasible. However, further investigation into strategies to augment *in vivo* NK cell persistence and expansion are needed.

Future efforts generating novel NK cell products for adoptive transfer are likely to be investigated in the ovarian cancer setting. *Ex vivo* inhibition of GSK3 kinase in peripheral blood enhances CD57 expression and late-stage maturation of NK cells (176). These NK cells demonstrated significantly higher production of cytokines (TNF-α and IFN-γ) and ADCC when exposed to cancer cells. Recruitment for an ovarian cancer clinical trial using the product generated from this method has opened at the University of Minnesota. Another recent study evaluated the potency of NK cells derived from human CD34^+^ hematopoietic stem and progenitor cells (HSPC) against a mouse xenograft model for ovarian cancer (177). Mice that received IP HSPC-NK cell infusions had significantly reduced tumor progression compared to controls. Finally, efforts to generate ovarian cancer specific NK chimeric antigen receptors are underway (178). These engineered proteins consist of a fused single-chain variable fragment (scFv) to an intracellular signaling domain to enhance NK effector function. A combination of these different techniques of generating NK cell products hold great promise and may make IP adoptive transfer effective against ovarian cancer following primary cytoreductive surgery and adjuvant chemotherapy.

### Other Immunotherapeutic Options to Enhance NK Cell Function

Other immunotherapeutic strategies are currently being characterized for antitumor activity (162). The development of drugs known to influence NK cell presence and function include mAb therapy, immunomodulatory drugs, vaccines (peptide, viral-based, tumor antigens, dendritic cells), and the adoptive transfer of T cells, dendritic cells, and macrophages. Even commonly used cytotoxic agents increase expression of NK cell-activating ligands and enhance NK cell recognition and killing more than others (179). A thorough discussion of each method is outside the scope of this review, but we will comment on several key issues.

Antibody-based immunotherapy has transformed the treatment of many malignancies, but is not yet standard of care for ovarian cancer. The mAbs function through two separate mechanisms. First, treatment is aimed at antigens present on tumor cells to facilitate an antitumor response through opsonization and activation of ADCC. Several tumor-associated antigens targeted with mAb for ovarian cancer have been identified, including NY-ESO-1, CA 125 (MUC16), MUC1, and epithelial cell adhesion molecule (EpCAM) (154). mAbs can also function in a non-immune-mediated manner to block vital growth and survival pathways, such as Her2/Neu, membrane folate receptor, and VEGF. Clinical trials evaluating the efficacy of mAbs should include investigations into both mechanisms.

A newer approach involves engineered bispecific antibodies and bispecific/trispecific killer engagers (BiKEs or TriKEs), which are molecules that cross-link antigens on tumor cells with CD16 on NK cells, activating and enhancing ADCC (180). One example utilized anti-CD16 scFv spliced to anti-EpCAM scFv (181). This BiKE promoted immune synapse and ADCC between NK cells and EpCAM-expressing tumor cells. More recently, a fully humanized TriKE utilized a modified IL-15 to cross-link the anti-CD16 scFv and EpCAM scFv (182). The 1615EpCAM TriKE was specific and active against EpCAM bearing ovarian cancer cells and mediated NK proliferation, sustained ADCC activity, improved lytic degranulation, and cytokine production. A TetraKE construct incorporating the cancer stem cell marker anti-CD133 scFv was recently engineered to simultaneously target EpCAM and CD133 bearing cells (183). These engineered small molecules combine the specificity of mAbs with the NK cell expansion and survival benefits of cytokine therapies *via* IL-15 into a single product. This novel strategy to target NK cells for antigen-specific immunotherapy has recently been reviewed and will hopefully prove effective in supplementing traditional therapies against gynecologic malignancies (184, 185).

Immune checkpoints are inhibitory pathways that serve to prevent self-tissue damage. During tumorigenesis, cancer cells often express ligands to bind and induce immune suppression. A number of antibodies have been developed to block checkpoint pathways expressed on certain T cells, B cells, monocytes, and NK cells, including CTL-associated protein 4, programmed death protein 1 (PD-1), TIM-3, NKG2A/CD94 complex, and CD96/CD226/TIGIT receptors (186, 187). A recent publication identified a population of NK cells within the ascites of women with ovarian cancer where PD-1 is highly expressed suggesting therapies targeting PD-1/PD-L may be effective (188). In fact, *in vitro* studies have shown that PD-1 and CD96/TIGIT blockade augments NK cell-mediated tumor lysis (189, 190). Future research is needed to clarify the effects checkpoint inhibitors have on the NK cell response and the potential to enhance adoptive NK cell immunotherapy.

## Concluding Remarks

Here, we provide an extensive overview of NK cell-based immunobiology and therapy in gynecologic malignancies. Over the past several decades, insight into biology controlling activation or inhibition has advanced the prospect of NK cell-based immunotherapy, which is just now being realized. Today, there are strategies to harness NK cell function for immunotherapy, including: (i) adoptive transfer of alloreactive NK cells, (ii) blocking NK inhibitory signals with mAb, (iii) promoting death ligand expression, and (iv) enhancing specificity *via* activation of ADCC. In addition, using drugs or cytokines to promote NK cell proliferation and function or inhibit NK cell suppressors are potential strategies. Complementary approaches also exist to manipulate the genetics geared to maximize NK cell function against specific tumor targets. One example includes viral transduction and gene transfection through electroporation technologies with the goal of increasing production of cytokines or cell receptors (191).

There are several crucial issues that require consideration for adoptive NK cell-based cancer immunotherapy that need to be highlighted. These include (i) standardizing protocols and techniques in NK cell preparation, (ii) establishing firm criteria for donor selection to improve clinical response, (iii) identifying the best method of conditioning recipients to avoid rejection and promote survival of transferred NK cells, (iv) combining NK cell-based immunotherapy with other therapies to eliminate cancer cells, and (v) enhancing understanding of tissue immunity and the tumor microenvironment (3).

Finally, early clinical studies have demonstrated promise of NK cell-based immunotherapy for gynecologic malignancies. Future research will be important to identify patients that will most likely benefit from immunotherapy and define the specific role and timing of therapy. In addition, combination approaches need to be explored and optimized before therapeutic breakthroughs can realistically be envisioned.

## Author Contributions

LU and CD wrote the manuscript with contributions from MF and MG. JM, MF, and MG edited the manuscript.

## Conflict of Interest Statement

JM reports the following scientific advisory boards: (1) Celgene (2) Fate Therapeutics, and (3) GT BioPharma. He also receives research funding from Fate Therapeutics and GT BioPharma. The remaining coauthors declare that the research was conducted in the absence of any commercial or financial relationships that could be construed as a potential conflict of interest.
